# Aldo-keto reductase 1B10 as a Carcinogenic but Not a Prognostic Factor in Colorectal Cancer

**DOI:** 10.7150/jca.91064

**Published:** 2024-01-27

**Authors:** Xu Ye, Tao Wang, Liyuan Zhong, Jaume Farrés, Jiliang Xia, Xi Zeng, Deliang Cao

**Affiliations:** 1 Hunan Cancer Hospital and the Affiliated Cancer Hospital of Xiangya School of Medicine, Central South University, Hunan 410031, China.; 2 Hunan Province Key Laboratory of Cancer Cellular and Molecular Pathology, Cancer Research Institute, University of South China Hengyang Medical College. 28 W Changsheng Road, Hengyang, Hunan 421009, China.; 3 Department of Biochemistry and Molecular Biology, Universitat Autònoma de Barcelona, E-08193, Bellaterra, Barcelona, Spain.

**Keywords:** Colorectal cancer, AKR1B10, Biomarker, Tissue microarrays, and DNA damage

## Abstract

Colorectal cancer (CRC) is the leading cause of cancer death, but little is known about its etiopathology. Aldo-keto reductase 1B10 (AKR1B10) protein is primarily expressed in intestinal epithelial cells, but lost in colorectal cancer tissues. This study revealed that AKR1B10 may not be a prognostic but an etiological factor in colorectal tumorigenesis. Using a tissue microarray, we investigated the expression of AKR1B10 in tumor tissues of 592 colorectal cancer patients with a mean follow-up of 25 years. Results exhibited that AKR1B10 protein was undetectable in 374 (63.13%), weakly positive in 146 (24.66%), and positive 72 (12.16%) of 592 tumor tissues. Kaplan-Meier analysis showed that AKR1B10 expression was not correlated with overall survival or disease-free survival. Similar results were obtained in various survival analyses stratified by clinicopathological parameters. AKR1B10 was not correlated with tumor T-pathology, N-pathology, TNM stages, cell differentiation and lymph node/regional/distant metastasis either. However, AKR1B10 silencing in culture cells enhanced carbonyl induced protein and DNA damage; and in ulcerative colitis tissues, AKR1B10 deficiency was associated acrolein-protein lesions. Together this study suggests that AKR1B10 downregulation may not be a prognostic but a carcinogenic factor of colorectal cancer.

## Introduction

Colorectal cancer (CRC) is one of the most common cancers worldwide; lifetime risk of developing this malignancy is up to 6% of population 1. Colorectal cancer is preventable by endoscopic screening and removal of pre-malignant adenomatous polyps, from which most colorectal cancer in the average-risk population develop 2, 3. Therefore, prevention is often a definitive therapeutic procedure and is cost-effective and life-saving, but the compliance is a challenge in most patients as the painful gastrointestinal preparations. In addition to adenomas, ulcerative colitis (UC), a chronic inflammatory bowel disease (IBD), is a predisposing condition of colorectal cancer, usually called colitis-associated colorectal cancer (CAC). In UC patients, the risk of developing colorectal cancer is up to 40%, and the risk exponentially rises with duration of the disease 4, 5. However, the current estimates of colorectal cancer risk are largely based on retrospective studies; the molecular etiopathology remains to be understood.

Aldo-keto reductase 1B10 (AKR1B10), also known as aldose reductase-like-1 (ARL-1), is a secretory protein identified in human hepatocellular carcinoma (HCC) 6-8. AKR1B10 is a NADPH-dependent monomeric enzyme with strong enzymatic activity toward electrophilic carbonyl compounds 9-12. Electrophilic carbonyl compounds are highly reactive aldehydes and ketones that interact with cellular nucleophiles and form covalently modified protein- and/or DNA-adducts 13-16. These non-specific carbonyl modifications on proteins may cause protein dysfunction, resistance to cellular proteolysis, and depolymerization; the carbonyl-nucleotide adducts may block DNA semiconservative replication, arrest gene transcription, and induce DNA mutations and breaks 15, 17, 18. Therefore, reactive carbonyl compounds are highly cytotoxic, mutagenic and carcinogenic 15, 19. AKR1B10 catalyzes reduction of the reactive carbonyl group into corresponding alcoholic group, protecting host cells from carbonyl lesions. In addition, as a NADPH-dependent reductase, AKR1B10 is well accepted as a critical enzyme in retinoid metabolism, converting all-trans-retinaldehyde to retinol 20, 21. This process may exhaust the cellular retinoic acid, which is crucial to cell proliferation and differentiation 21, 22, and makes AKR1B10 as a potential target for development of small chemical inhibitors^23^, ^24^.

AKR1B10 is also a key regulator of long-chain fatty acid and lipid synthesis in cells. AKR1B10 binds with acetyl-CoA carboxylase-α (ACCα), a rate-limiting enzyme in *de novo* synthesis of long-chain fatty acids, and prevents its ubiquitination and proteasomal degradation, thus promoting *de novo* synthesis of fatty acids 25. Long-chain fatty acids are essential components of cellular lipids, including membrane phospholipids for cell division and signaling transduction 26-28. AKR1B10 silencing leads to decrease of membrane lipid messenger diacylglycerol (DAG) and inhibition of the PKC/ERK signaling pathway 29.

AKR1B10 is primarily expressed in normal colon and small intestine with a detectable level in liver 6, 30. AKR1B10 is not expressed in normal breast, lung and pancreas tissues, but upregulated in tumors developed from these organs, including breast cancer 31, pancreatic cancer 32, lung squamous cell carcinomas and adenocarcinomas 33, and hepatocellular carcinoma 6, 34, 35. In these tumors, AKR1B10 functions as an oncogenic protein and promotor of tumor growth and progression, which may be ascribed to its biological function in electrophilic carbonyl elimination, retinoic acid metabolism and fatty acid/lipid synthesis as discussed above, and also as a diagnostic and prognostic marker of cancer 34, 36. However, it is a different scenario in the intestine, where AKR1B10 is normally expressed, but undetectable or markedly diminished in UC, Crohn's disease and colorectal cancer tissues 37-39. In mice, targeted deficiency of AKR1B8 (an orthologue of human AKR1B10) leads to high susceptibility to dextran sulfate sodium (DSS)-induced colitis and associated colorectal tumorigenesis 39; in humans, AKR1B10 was reported as a negative prognostic marker of colorectal cancer 40. This study evaluated AKR1B10 expression in 592 colorectal cancer cases with a mean follow-up of 25 years and evaluated its potential role in development and prognosis of colorectal cancer. Our data proposed a novel paradigm of AKR1B10 in colorectal cancer.

## Materials and Methods

### Frozen tissues, tissue microarrays and clinical data

Surgically resected frozen specimens were collected with an approved IRB protocol. All specimens were quality-controlled by pathologists. Tissue microarray (TMA) of colorectal cancer was obtained from Tissue Microarray Facility, Department of Pathology at Yale University, which represented a collection started from 1960s. All cases have entire pathological and follow-up data. This TMA consisted of 619 colorectal cancers, 10 normal colons, and 10 cultured cells. Additionally, ten colorectal cancer tissues were randomly duplicated and sporadically spread in this TMA for a quality control.

### Cell culture and AKR1B10 silencing by siRNA

HCT-8 and A549 cells, purchased from American Type Culture Collection (Manassas, VA), were maintained in RPMI-1640 or DMEM medium (Hyclone, UT) containing 10% fetal bovine serum (FBS), 2 mM glutamine, 100 U/ml penicillin, and 100 µg/ml streptomycin at 37^o^C, 5% CO_2_. *AKR1B10* silencing was conducted as described previously 10. Briefly, cells (3.5 x 10^4^ in Opti-MEM I medium) were mixed gently with small-interfering RNA (siRNA) and OligofectAMINE (Invitrogen, CA) in a volume of 0.5 ml following manufacturer's instructions, and then incubated at 37^o^C, 5% CO_2_ for 4 hours, followed by addition of an equal volume of fresh medium containing 20% FBS. Cells were continuously cultured until harvest.

### Western blot

Culture cells were lysed in lysis buffer (Roche, IN) and centrifuged at 12,000*g* for 10 min to collect soluble proteins. Tissues were homogenized on ice in 300µl of 50mM Tris.Cl buffer (pH 7.0), followed by centrifugation at 12,000*g* for 10 min to collect supernatants. For Western blot, proteins (30 ~ 50 µg) were separated on 12% SDS-PAGE and blotted onto a pure nitrocellulose membrane (Bio-Rad, CA) at 180 mA for 2 hours. After being blocked with 5% skim milk in PBS at room temperature for 45 min, membranes were incubated with AKR1B10 (1:500) or acrolein (1: 500, Advanced Targeting System, CA) antibodies at 4^o^C overnight, followed by incubation with goat anti-rabbit or anti-mouse IgG (1:2000; Sigma, MO) for 1 hour. Antibody binding signals were detected using enhanced chemiluminescence system (Pierce, IL). Protein loading amount was normalized by re-probing the membrane with β-actin monoclonal antibody (1:40,000; Sigma, MO).

### DNA damage assays

DNA mutations were examined by hypoxanthine-guanine phosphoribosyl transferase (HPRT) mutant selection with 7.0 mg/ml 6-thioguanidine (6-TG) for 10-12 days. Briefly, cells were spread at 1-5 x 10^5^ cells per 60mm dish and exposed to 6-TG; colonies were then stained with 1% crystal violet for counting and photographing. DNA breaks were examined by comet assays as described by Singh, et al. 41. Isolated cells were analyzed with TriTek Cometscore version 1.5.

### Immunohistochemistry and immunocytochemistry

Immunohistochemistry of TMA was initiated by deparaffinizing and rehydrating as below: two times for 10 min in xylene, once for 2 min in 100% ethanol, once for 2 min in 90% ethanol, and once for 2 min in 70% ethanol. In a Coplin jar with antigen retrieval buffer (10 mM citric acid, pH 6.0), TMA was completely immersed and microwaved (at 700 watts) for 5-20 min. After cooling for 20 min at room temperature (RT), TMA was rinsed in deionized water two times for 5 min each. Endogenous peroxidase was inactivated with 0.3% H_2_O_2_ at room temperature for 20 min. Incubation with anti-AKR1B10 was performed at 4°C overnight. After being washed by PBS, the TMA was incubated with horseradish peroxidase conjugated secondary antibody (1:100) at 37°C for 2 hours, followed by chromogen 3,3'- diaminobenzidine (DAB) detection of peroxidase at 37°C for 10-30 seconds. Thereafter, the TMA was counter-stained with hematoxylin to indicate nuclei and mounted with a cover slide 31. AKR1B10 expression was evaluated independently by a researcher and a pathologist and scored as follows: negative (not detectable), weakly positive (+1, low-intensity of AKR1B10 staining), and positive (+2, high-intensity of AKR1B10 staining).

For immunocytochemistry, cells cultured on cover slides were fixed in ice-cold methanol for 10 min and ice-cold acetone for additional 1 min. After exposed to 0.3% H_2_O_2_ at RT for 20 min, cells were incubated with anti-acrolein antibody (1:10) at 4^o^C overnight, followed by incubation with secondary antibody and DAB exposure as above.

### Statistical analysis

One-way analysis of variance (ANOVA) was used to compare AKR1B10 expression on age at diagnosis. Kruskal-Wallis tests were used to compare the groups on T-pathology, N-pathology and histological stages. Chi-square tests of independence, or exact tests as appropriate, were used to compare the groups of subjects with different histological grades and types and on their survival status. Kaplan-Meier survival curves were computed to estimate the survival of patients. Curves were compared using the log-rank test. Results were considered statistically significant for *p*<0.05.

## Results

### AKR1B10 is downregulated in colorectal cancer

**Figure 1:**
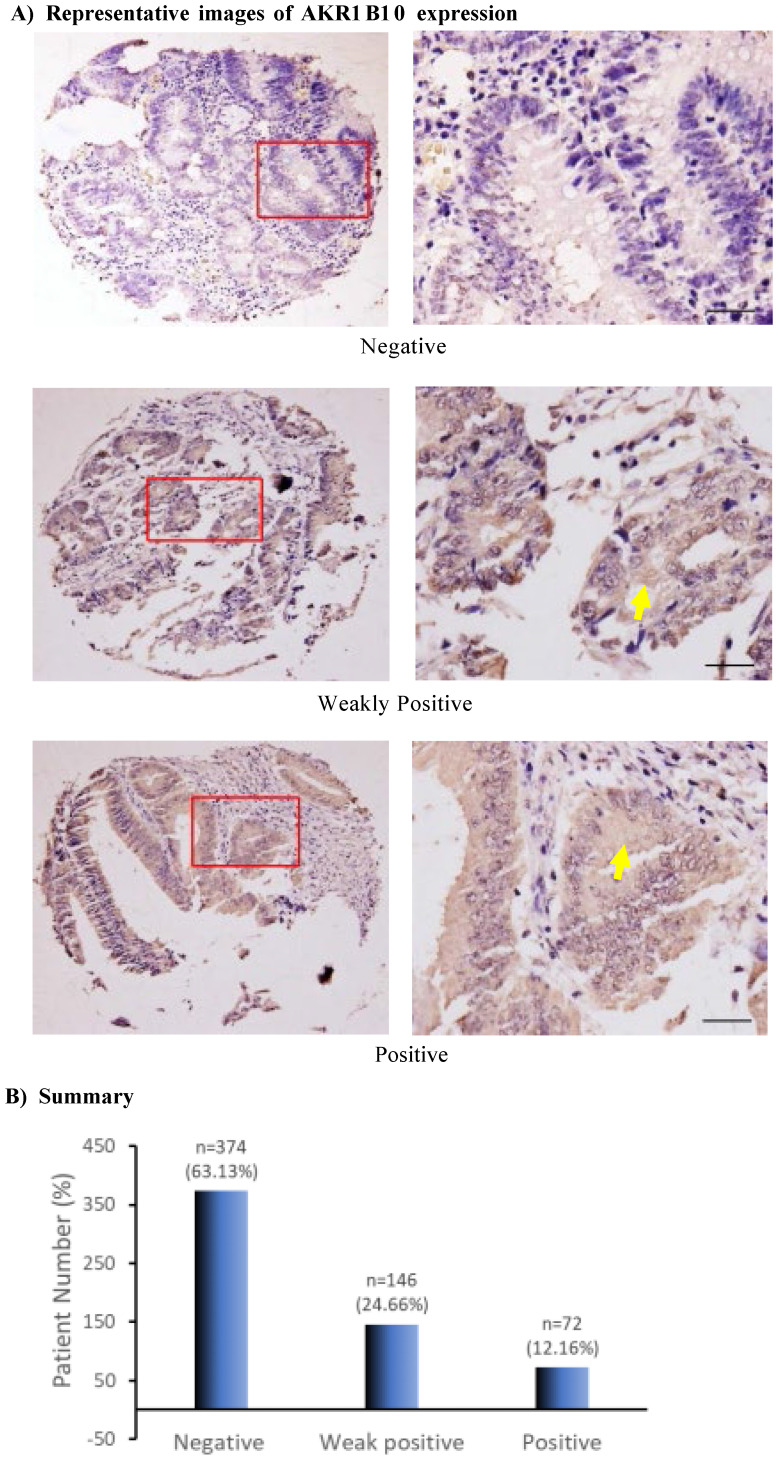

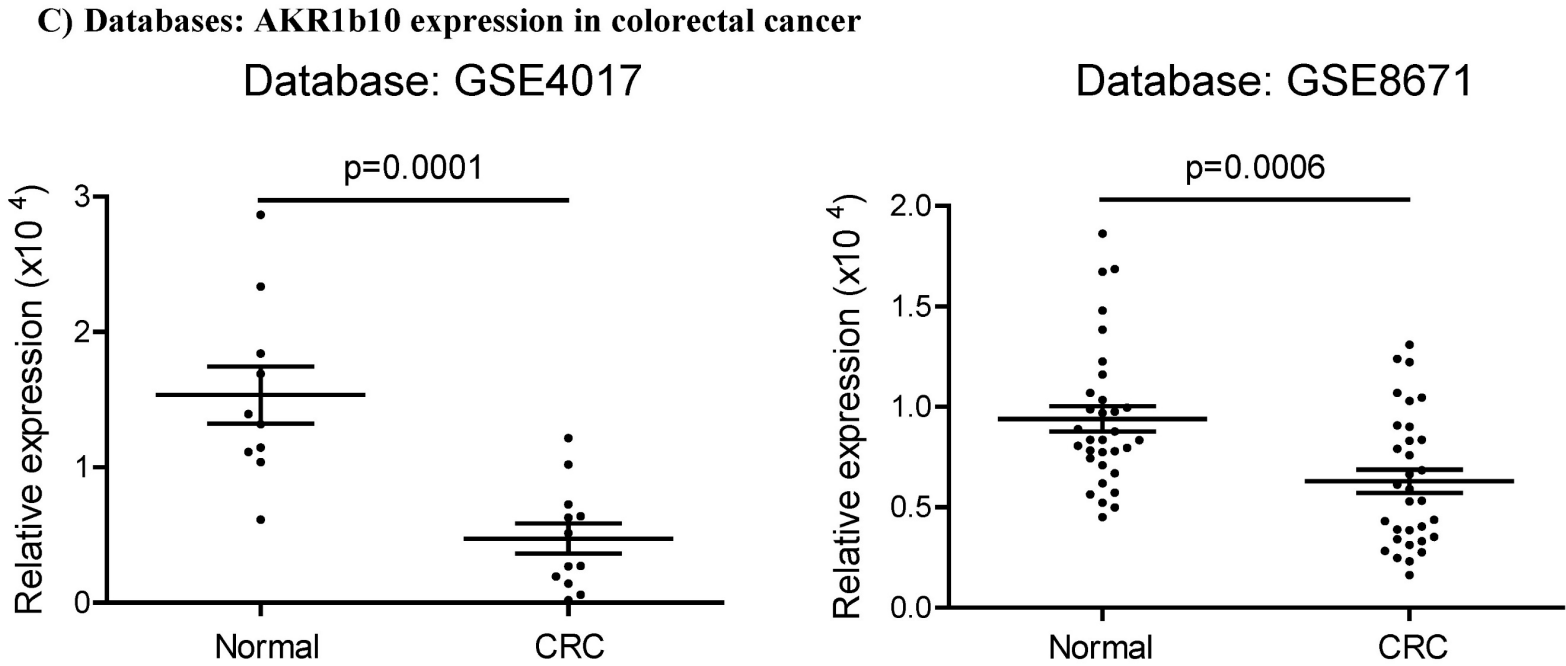
** AKR1B10 expression in colorectal cancer tissues.** Immunohistochemistry and evaluation of AKR1B10 protein expression were conducted as described in Materials and Methods. A) AKR1B10 expression at different levels. Data show the representative images. Arrows denote the weakly positive or positive staining. Scale bar, 50μm. B) Statistical summary of AKR1B10 expression in colorectal tissues. C) Databases: AKR1B10 expression in colorectal cancer. Data were from public microarray data sets, GSE4107 dataset (normal controls=10; CRC tissues=12) and GSE8671 dataset (normal controls=32; CRC tissues=32).

This study evaluated AKR1B10 expression in colorectal cancer using a tissue microarray that contained 619 cases with complete pathological and follow-up data of 25 years. After evaluation by two independent investigators, a total of 592 tissue spots from different patients showed quality of histology and immunohistochemistry and were scored for AKR1B10 expression at negative (0), weakly positive (+1) or positive (+2). Out of 592 cases, AKR1B10 was not detected (negative) in 374 (63.18%) cases, weakly positive in 146 (24.66%) cases, and positive in 72 (12.16%) cases (Figure 1), indicating the downregulation of AKR1B10 in a large number of colorectal cancer tissues. AKR1B10 downregulation was further confirmed by the data from public microarray datasets GSE4107 (normal controls=10; CRC tissues=12) and GSE8671 (normal controls=32; CRC tissues=32) (Figure 1C). A similar phenomenon was observed in the datasets of ulcerative colitis, GSE48958 (normal controls=8; UC tissues=13) and GSE14580 (normal controls=6; UC tissues=24) (Figure 4D), consistent with our previous report 39.

### AKR1B10 expression in colorectal cancer has no effects on patient survival

**Figure 2:**
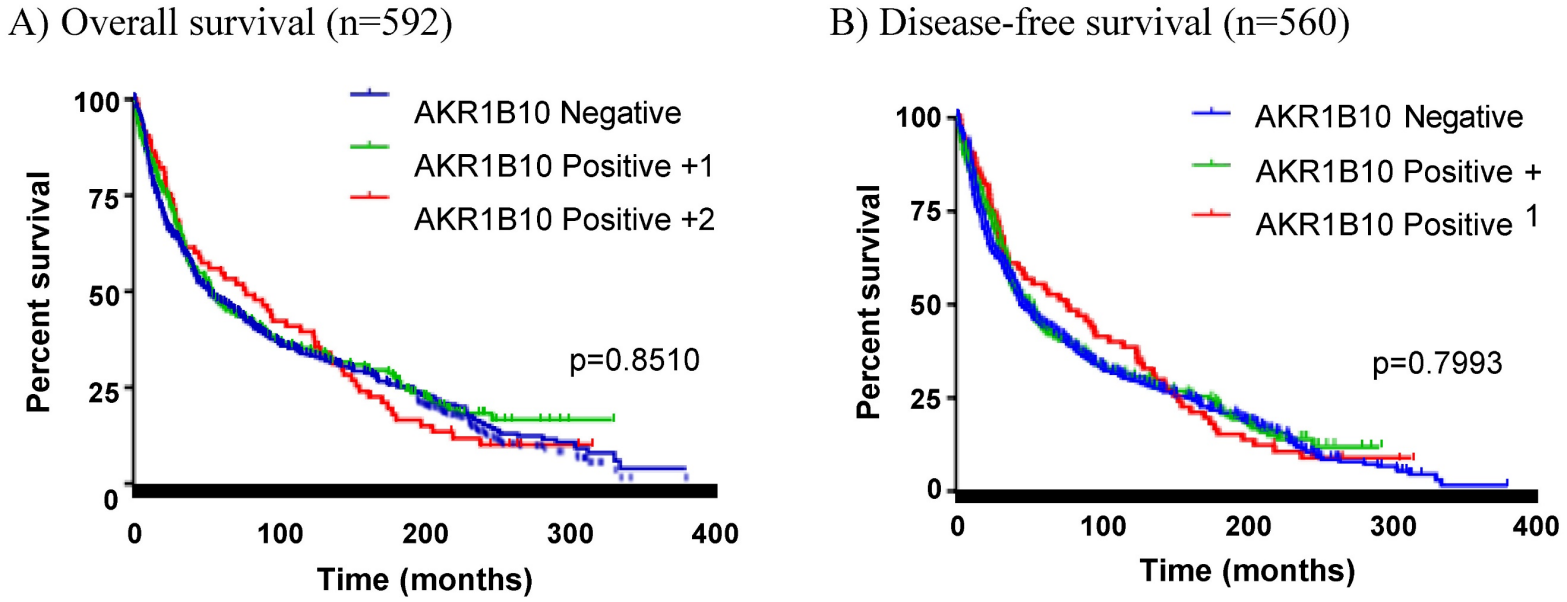
** Correlation between AKR1B10 expression and patient survival.** Patients were sorted into three groups: AKR1B10 negative, weakly positive (+1), and strongly positive (+2). Kaplan-Meier curves were used for survival analyses of patients. A) Overall survival; B) Disease-free survival.

**Figure 3:**
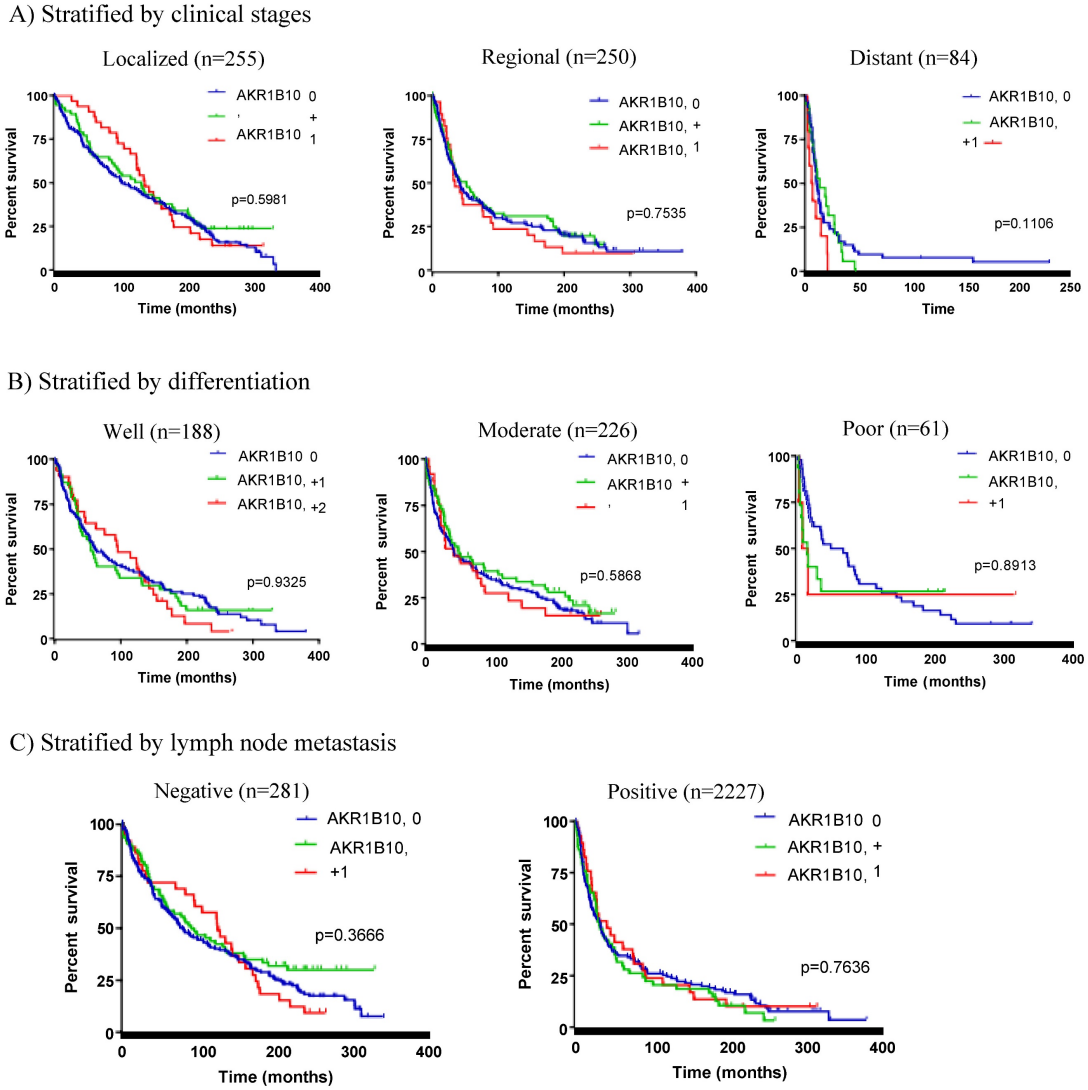
** Stratified survival analysis.** Patients were stratified by key clinicopathological factors and then subjected to Kaplan-Meier survival analyses. A) Stratified by clinical stages, B) Stratified by tumor cell differentiation, and C) Stratified by lymph node metastasis. AKR1B10 expression had not correlation with patient survival in any stratified analyses.

This cohort of 592 colorectal cancer patients represented a collection with a mean follow-up of 25 years, and thus we did a retrospective survival analysis with Kaplan-Meier. As shown in Figure 2, AKR1B10 expression was not correlated to the overall survival and disease-free survival of this cohort of colorectal cancer patients. We then conducted survival analyses stratified by clinicopathological parameters. As shown in Figure 3A, AKR1B10 expression was not associated with the survival of patients with a localized, regional or distantly metastatic tumor. Similarly, AKR1B10 expression was not associated with patient survival no matter of cancer cell differentiation (Figure 3B) and lymph node metastatic status (Figure 3C).

### AKR1B10 expression in colorectal cancer is not correlated with tumor types, pathological stages and lymph node and distant metastasis

We then evaluated whether AKR1B10 expression in colorectal cancer tissues was correlated with tumor growth and progression. As shown in Table 1, we assessed the correlation of AKR1B10 expression with various clinicopathological parameters; and results showed that the expression of AKR1B10 in colorectal cancer tissues was not associated with tumor types, T-pathology, N-pathology, TNM stages, cell differentiation, lymph node metastasis, and regional/distant metastasis. AKR1B10 expression was not correlated with patient age and gender, either. Taken altogether, our results suggest that AKR1B10 expression in colorectal cancer tissues has not any prognostic values.

### AKR1B10 deficiency leads to acrolein-induced protein damage in culture cells and UC tissues

**Figure 4:**
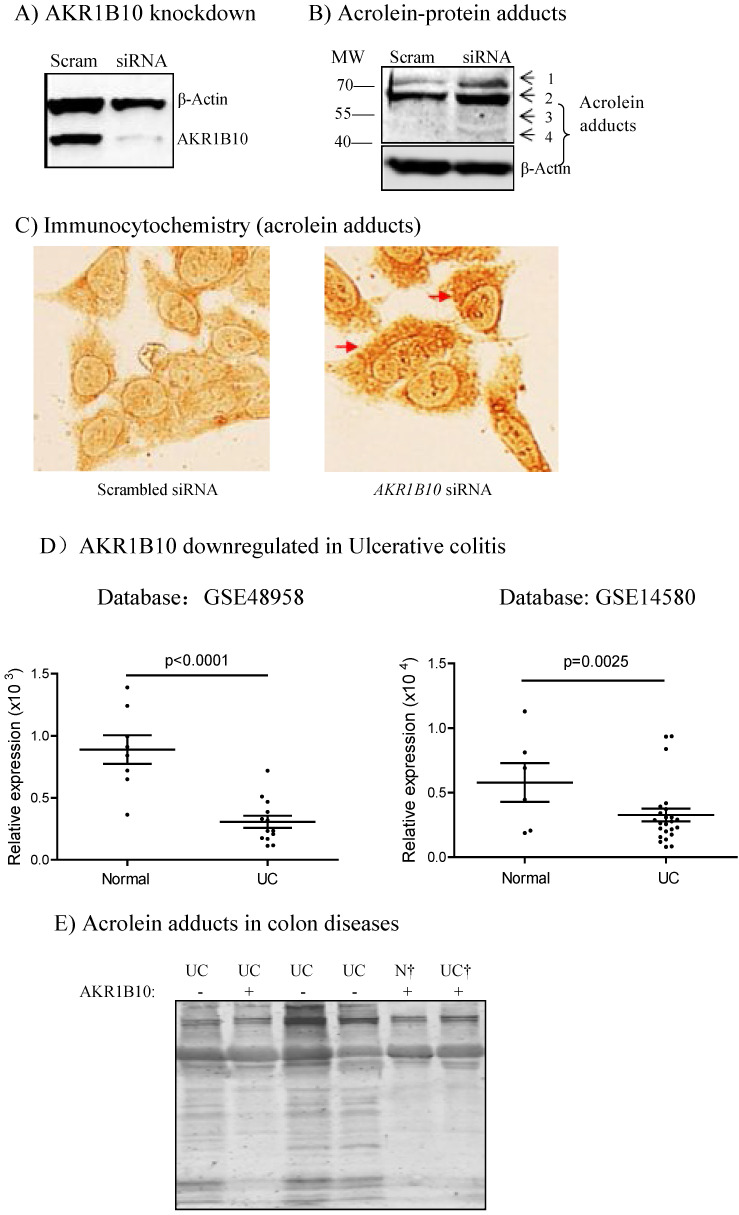
** Acrolein-protein lesions induced by AKR1B10 deficiency.** AKR1B10 silencing, Western blot, and immunocytochemistry were conducted as described in Materials and Methods. A) siRNA-mediated AKR1B10 silencing. B) Western blot for acrolein-adducted proteins in AKR1B10 silencing cells. C) Immunocytochemistry for acrolein-adducted proteins in AKR1B10 silencing cells. Arrows denote acrolein adducts of proteins in the cells. D) AKR1B10 expression in. Data were from public microarray datasets, GSE48958 dataset (normal controls=8; UC tissues=13) and GSE14580 dataset (normal controls=6; UC tissues=24). E) Western blot for acrolein-adducted proteins in ulcerative colitis tissues. N, normal tissue; UC, ulcerative colitis. †, paired specimens.

AKR1B10 can efficiently eliminate cellular carbonyl compounds at physiological levels, which are highly cytotoxic 9. We thus evaluated carbonyl-induced protein damage. Acrolein is a risk factor of colorectal cancer and AKR1B10 catalyzes reduction of acrolein to a less cytotoxic alcoholic form 42-44. Therefore, acrolein-protein adducts were evaluated as a representative of carbonyl-induced protein damage in cells. Results showed that silencing of AKR1B10 in HCT-8 cells by siRNA (Figure 4A) led to marked increase of acrolein-protein adducts (Figure 4B & C). In AKR1B10 knockdown cells, bands 1 and 2 were stronger than those in the scrambled control, and two additional bands 3 and 4 appeared. The accumulation of acrolein adducts was also validated in A549 cells after AKR1B10 was knocked down (Figure S1). AKR1B10 is downregulated in UC tissues (Figure 4D), and thus we further examined the acrolein-protein adducts in UC tissues. As shown in Figure 4E, acrolein-protein adducts were notably enriched in UC tissues without AKR1B10 expression. These data indicate that AKR1B10 protects host cells from acrolein-induced protein lesions.

### AKR1B10 deficiency leads to DNA breaks and mutations

**Figure 5:**
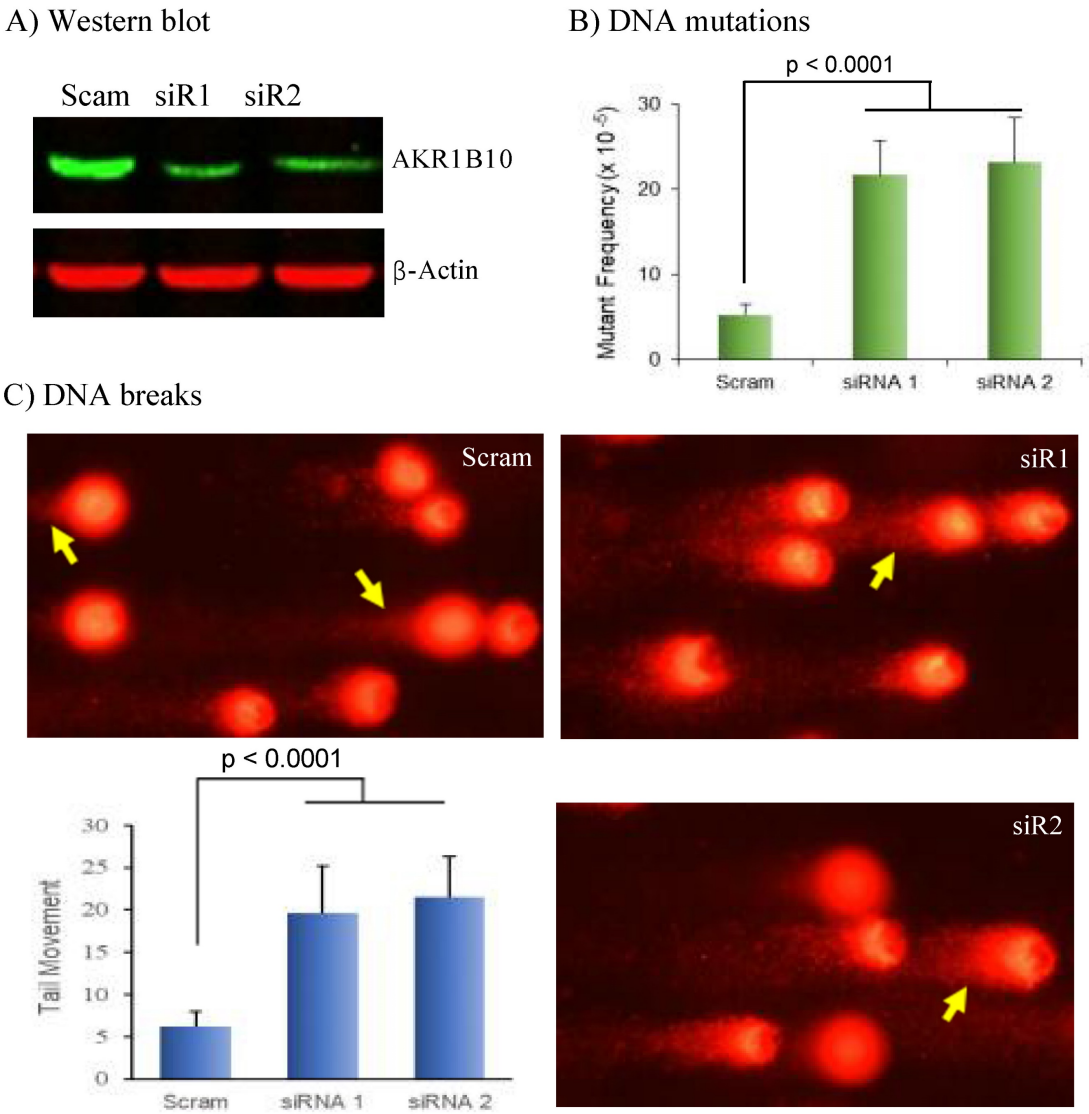
** DNA damage induced by AKR1B10 deficiency.** AKR1B10 silencing, DNA mutation assays, and comet assays were conducted as described in Materials and Methods. A) siRNA-mediated AKR1B10 silencing. B) DNA mutations by HPRT selection in AKR1B10 silencing cells. C) DNA breaks by comet assays in AKR1B10 silencing cells. HPRT, hypoxanthine-guanine phosphoribosyl transferase. Arrows denote comet tails of damaged DNA.

Electrophilic carbonyl compounds are highly genotoxic 45, 46. We thus further evaluated the effect of AKR1B10 silencing on genome stability. DNA mutations induced by AKR1B10 silencing were estimated by HPRT mutation assays 41. As shown in Figure 5B, the HPRT mutant frequency was significantly increased in HCT-8 cells with AKR1B10 silencing compared to that in vector control cells. Comet assays for the assessment of DNA breaks showed that AKR1B10 silencing led to significant increase of comet tail movements in HCT-8 cells (Figure 5C), which was further validated with A549 cells (Figure S2). These data indicate that AKR1B10 may be an important protein for protection of the genome DNA from carbonyl lesions.

## Discussion

### AKR1B10 is downregulated in colorectal cancer but has not prognostic values

AKR1B10 is specifically expressed in normal colon epithelial cells, but downregulated in colorectal cancer and considered as a negative prognostic marker 6, 37, 40. This study extensively evaluated the expression and prognostic value of AKR1B10 in a cohort of 592 colorectal cancer cases with a mean follow-up of 25 years. AKR1B10 was downregulated in 87.79% of colorectal cancer (undetectable in 63.13% cases and diminished in 24.66%), which is consistent with literature reports 37-39. However, retrospective analysis of the database indicated that AKR1B10 downregulation in colorectal cancer was not correlated with tumor types, histological stages, cell differentiation and lymph nodes and distant metastasis; AKR1B10 expression in colorectal cancer did not have effects on overall and disease-free survival and in various survival analysis stratified by clinicopathological parameters. Therefore, AKR1B10 deficiency in colorectal cancer tissues is not a prognostic marker, disagreeing with a previous literature report 40.

### AKR1B10 promotes cell proliferation and survival, but its tumor-promoting role is organ-dependent

The essential biological function of AKR1B10 is elimination of carcinogenic carbonyl compounds and promotion of lipogenesis, thus promoting cell proliferation and survival. In breast cancer, AKR1B10 is upregulated in ductal carcinoma *in situ*, infiltrating carcinoma and recurrent cancer, and correlated with tumor size, lymph node metastasis, and worse survival, being a negative prognostic factor 31. In breast cancer cells, AKR1B10 expression enhances cellular levels of important lipid second messengers, such as phosphatidylinositol bisphosphate (PIP2), diacylglycerol (DAG) and inositol triphosphate (IP3), and thus activates PKC/ERK signaling cascade, promoting tumor growth 29. In breast cancer, AKR1B10 also promotes cell invasion and metastasis through stimulation of the FAK/Src/Rac1 signaling pathway 47. In lung cancer, AKR1B10 is also induced and promotes cancer cell proliferation and metastasis, being a negative prognostic factor 48. Similarly, AKR1B10 activates K-Ras mediated MEK/ERK signaling activity in pancreatic cancer 32.

AKR1B10 is also upregulated in hepatocellular carcinoma (HCC), but its role in progression and prognosis of HCC is controversial. In preclinical models, targeted expression of AKR1B10 promotes HCC cell growth and proliferation 35, but studies on clinical samples of HCC indicated that AKR1B10 was upregulated in well differentiated and early-stage tumors and thus was proposed as a positive prognostic marker 49. In contrast, AKR1B10 was also proposed as a negative prognostic factor of HCC 50. Further study is warranted.

Opposite to these cancer types discussed above, AKR1B10 is downregulated in colorectal cancer, and this study did not support its prognostic value. These differential roles of AKR1B10 in different types of cancers may be ascribed to the intrinsic context of organs, rather than to its biological functions.

Colonic epithelial cells are constantly self-renewed, from stem cells at the bottom, to progenitor cells in proliferating zone, and then to mature cells at apical face where matured epithelial cells are apoptotic and shed into the lumen. The half-life of colonic epithelial cells is 2-3 days in mice and 6-7 days in humans 51, 52. Colon epithelial cells thus have high needs of lipids for cell division and lipid messenger-mediated signaling transduction. Colon epithelial cells are also constantly exposed to various carbonyl compounds derived from daily food consumptions and active microbial and cellular metabolism 53-55. Therefore, AKR1B10 is expressed in epithelium lining the colon for lipid synthesis and cellular protection from carbonyl lesions. AKR1B10 deficiency in epithelial cells leads to insufficiency of lipids for cell division and signal transduction and to exposures to carbonyl stress, which makes the epithelial cells vulnerable to various pathogenic factors and carcinogenesis. In fact, AKR1B8 deficient mice are highly susceptible to DSS-induced colitis and colitis-associated tumorigenesis, supporting this hypothesis 39.

In sharp contrast, normal mammary and lung bronchial epithelial cells are not self-renewed, with low lipid needs and carbonyl stress. While being transformed, the cells face challenges of lipid needs and carbonyl stress due to enhanced cell proliferation and metabolism, in which induced AKR1B10 protects cells from carbonyl lesions and promotes lipogenesis for cell division and signaling transduction. Therefore, AKR1B10 is upregulated in breast and lung cancers and promotes tumor progression and worse prognosis; and AKR1B10 may play a tissue-specific role in the tumorigenesis and cancer progression (Figure S3).

### AKR1B10 deficiency may drive carcinogenic transformation of colon and rectum, particularly in UC-associated tumorigenesis

This study indicates that AKR1B10 deficiency may not be a prognostic but a potential carcinogenic factor in colorectal tumorigenesis through induction of carbonyl stress and cellular lesions. This opinion is supported by a few lines of evidence from our and other laboratories. First, AKR1B10 deficiency induces carbonyl-DNA and carbonyl-protein damages. Electrophilic carbonyl compounds are carcinogenic factors 15, 16, 42. Reactive carbonyls possess the capability to induce DNA damage through a mechanism known as DNA glycation, forming DNA adducts. This process is cytogenic and carcinogenic through the increase of DNA mutation frequency and DNA strand breaks 56. Given its role in catalyzing the reduction of carbonyl compounds to less toxic alcoholic forms, it is proposed that AKR1B10 plays a pivotal role in preventing DNA damage induced by carbonyl compounds 57. In colonic cells, AKR1B10 deficiency leads to carbonyl-induced DNA mutations and breaks, as well as acrolein-protein adduct formation in culture cells and UC tissues. Importantly, intestinal epithelial cells constantly face carbonyl lesions by luminal microbial and cell metabolism, especially in oxidative stress condition of UC 18, 55. A worse scenario is that reactive carbonyl compounds are widely distributed in various diets and beverages 53, 54, 58. Therefore, AKR1B10 expressed in epithelial cells may serve as a “biochemical barrier” to eliminate reactive carbonyl compounds; AKR1B10 deficiency would make intestinal epithelial cells vulnerable to carbonyl lesions and malignant transformation.

Second, AKR1B10 deficiency leads to defects of intestinal epithelial cells in self-renewal and injury repair due to inhibition of lipid synthesis and signal transduction 29, 39. Silencing of AKR1B10 leads to decrease of cellular DAG and inhibition of PKC/ERK signaling activity 29. In mice, targeted disruption of AKR1B8, the orthologue of human AKR1B10 gene, leads to diminished proliferation, migration and maturation of colonic epithelial cells. AKR1B8 deficient mice are highly susceptible to DSS-induced colitis and colitis-associated tumorigenesis, suggesting the etiopathogenic role of AKR1B10 deficiency in UC and associated colorectal cancer 39.

Third, diminished AKR1B10 expression occurs in adenomatous polyps and UC tissues 59. Adenomatous polyps are precursors of colorectal cancer and approximately 80% of colorectal cancer arises from adenomatous polyps 60. UC is a predisposing condition with high risk of developing colorectal cancer 4; acrolein-protein lesions (Figure 4, S1) and DNA damage (Figure 5, S2) are increased in AKR1B10 silencing cells and in UC tissues with AKR1B10 deficiency. Therefore, AKR1B10 deficiency in the colon and rectum may be an early event and potential etiopathogenic factor in malignant transformation.

In summary, AKR1B10 is highly expressed in normal colon epithelium, where it protects the host cell from carbonyl lesions and promotes lipogenesis and signal transduction for self-renewal and damage repair. While AKR1B10 is deficient, epithelial cells face carbonyl-induced DNA and protein damage and also shortage of lipids for membrane synthesis and as second messenger molecules, thus inducing precancerous lesions and tumorigenesis. Therefore, AKR1B10 downregulation in colorectal cancer may not be a prognostic factor, but an early event and carcinogenic driver in colorectal carcinogenesis.

## Supplementary Material

Supplementary figures.

## Figures and Tables

**Table 1:** Correlation of AKR1B10 expression with clinicopathology and patient survival

	AKR1B10 Levels in Tumors (*n*=592)					
		Positive	Weakly Positive	Negative	*p* -Value^2^	
Age						
Mean		70.15	67.43	67.09	0.0952	
Median		71	68	68		
						
Sex						
Male		38	66	183	0.5517	
Female		34	80	191		
						
Tumor types						
Adenocarcinoma		65	136	334	0.2732	
M. adenocarcinoma		3	5	20		
Others		1	2	17		
						
T-pathology						
T0		1	0	2	0.4960	
T1		0	7	13		
T2		25	37	121		
T3		38	84	196		
T4		0	1	2		
						
N-pathology						
N0		35	79	176	0.9604	
N1		18	39	93		
N2		11	19	47		
						
Differentiation						
Well		31	47	110	0.2512	
Moderate		25	57	144		
Poor		4	15	42		
						
TNM Stages						
1		15	26	83	0.8679	
2		19	39	88		
3		27	58	141		
4		7	15	42		
						
Lymph Node Met						
No		38	78	178	0.6725	
Yes		34	68	156		
						
Metastasis						
Localized		33	57	164	0.7269	
Regional		29	69	151		
Distant		10	19	53		
						
Survival status						
Alive w disease		2	9	22	0.3621	
Alive w/o disease		7	21	34		
Dead w disease		30	68	176		
Dead w/o disease		33	48	142		

M, mucinous; Met, metastasis

## Abbreviations

AKR1B10: aldo-keto reductase 1B10
ARL-1: aldose reductase-like-1
CRC: colorectal cancer
DAB: chromogen 3,3'- diaminobenzidine
DAG: diacylglycerol
GAPDH: glyceraldehyde 3-phosphate dehydrogenase
HCC: hepatocellular carcinoma
IBD: inflammatory bowel diseases
NADPH: nicotinamide Adenine Dinucleotide Phosphate
TMA: tissue microarray
and UC: ulcerative colitis
