# Supplementary material for: Aldo-keto reductase 1B10 as a Carcinogenic but Not a Prognostic Factor in Colorectal Cancer
Source: J Cancer. 2024 Jan 27;15(6):1657–67. doi: 10.7150/jca.91064 (PMC10869966; doi:10.7150/jca.91064)
Supplement: Supplementary file 1 — Supplementary figures. [file jcav15p1657s1.pdf]

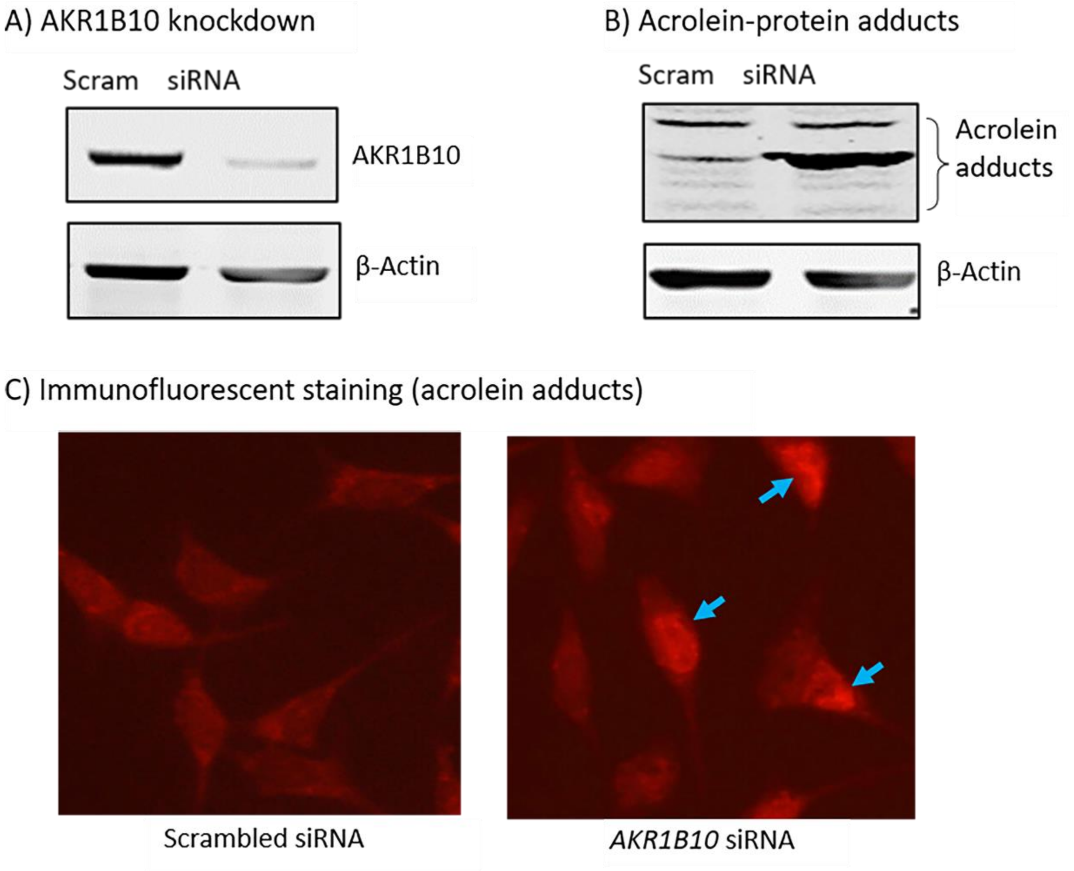

Figure S1. AKR1B10 knockdown induces acrolein-protein lesions in A549 cells. AKR1B10 was silenced as described in the manuscript for detection of acrolein-adducted proteins. A) siRNA-mediated AKR1B10 silencing; B) Westen blot for the detection of acrolein-adducted proteins; C) Immunofluorescence for acrolein-adducted proteins. Arrows denote the acrolein-adducts of proteins in the cells.

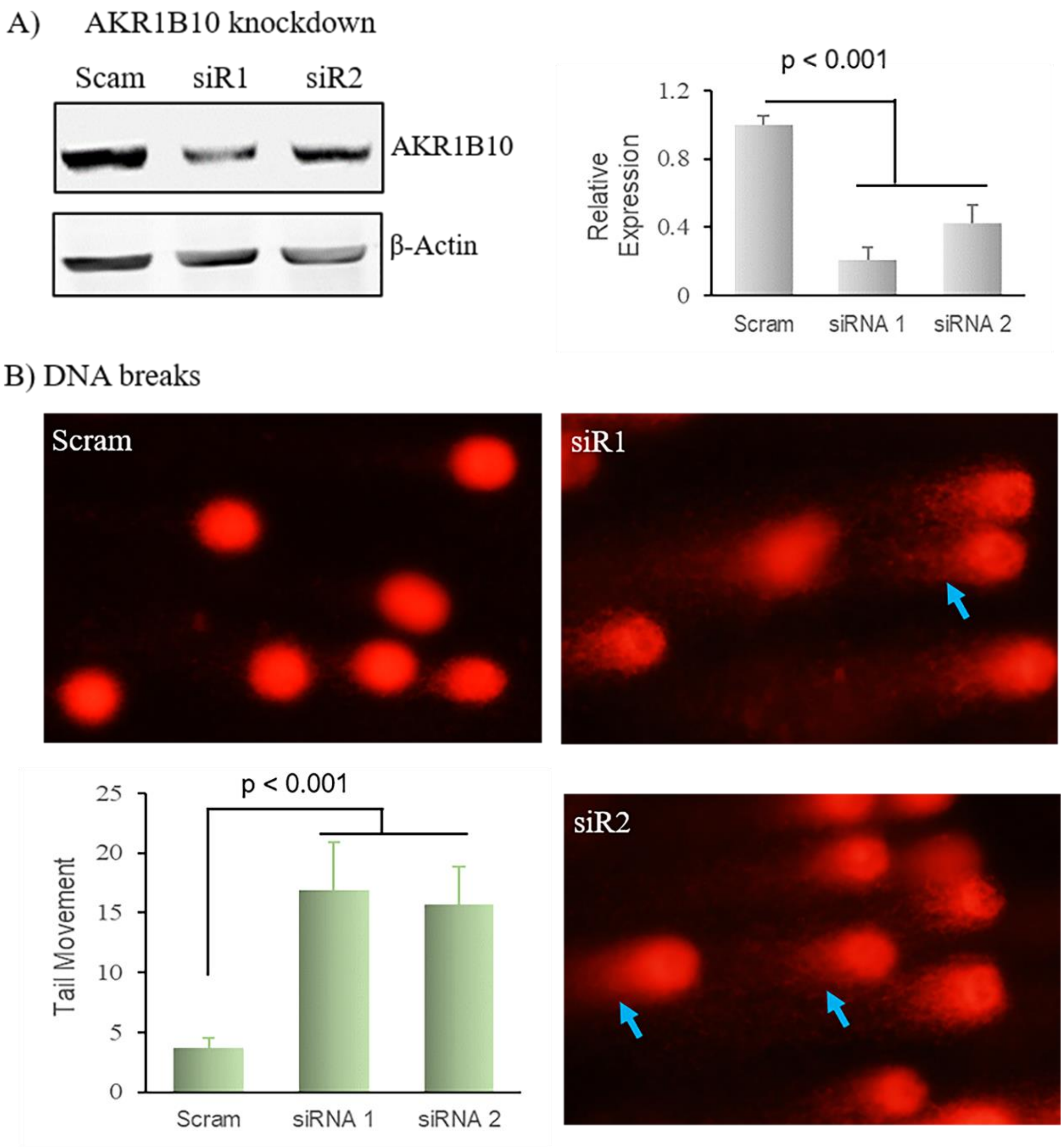

Figure S2. AKR1B10 knockdown induces DNA damages in A549 cells. AKR1B10 was silenced as described in the manuscript for detection of DNA damage. A) siRNA-mediated AKR1B10 silencing; B) DNA breaks by comet assays. Arrows denote comet tails of damaged DNA.

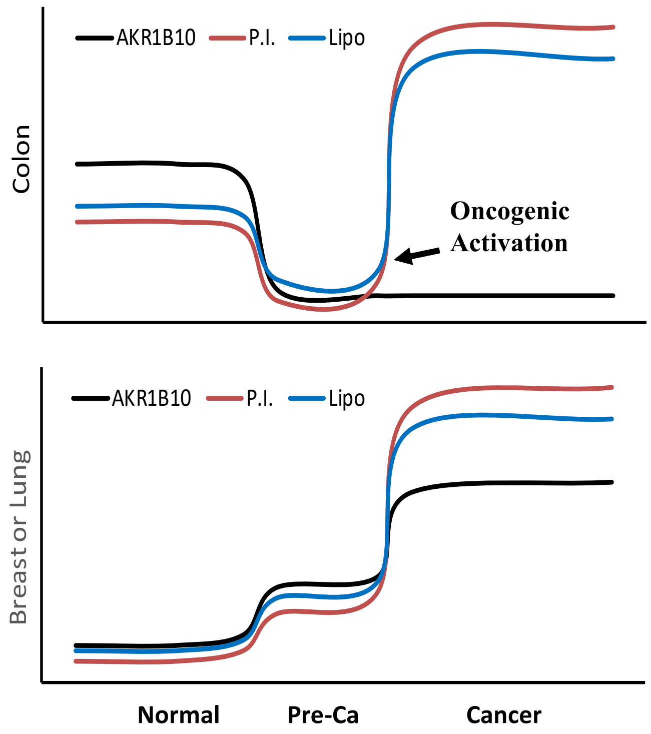

Figure S3. Hypothetic models of AKR1B10 in different organ-originated tumors. In colon (*upper panel*): normal colon epithelial cells are constantly self-renewed with high proliferation index (P.I.) and lipid needs. Colon epithelial cells also face constant carbonyl stress. Therefore, AKR1B10 is expressed for lipid synthesis (lipogenesis, Lipo) and cellular carbonyl elimination. AKR1B10 deficiency disrupts this hemostasis and thus leads to pre-cancerous lesions (Pre-Ca), such as colitis. Oncogenic activation (e.g., Ras mutations) that occurs in the pre-cancerous process may enhance lipogenesis and drive cell proliferation. In breast or lung (*lower panel*): normal mammary and bronchial epithelial cells are not self-renewed with low P.I., lipid needs, and carbonyl stress; AKR1B10 is not expressed in the normal cells. Oncogenic transformation of mammary and bronchial epithelial cells activates AKR1B10 expression, promoting cancer growth and progression.
